# RETRACTION: Legionella Pneumophila Infection Reduces the Mitochondrial Membrane Potential Through Lpg2444‐Inhibited Mitocytosis

**DOI:** 10.1111/cpr.13748

**Published:** 2024-09-15

## Abstract

JiC. , GaoJ. , and HuangY. , “Legionella Pneumophila Infection Reduces the Mitochondrial Membrane Potential Through Lpg2444‐Inhibited Mitocytosis,” Cell Proliferation (Early View): 10.1111/cpr.13660.38764239

The above article, published online on 19 May 2024 in Wiley Online Library (wileyonlinelibrary.com), has been retracted by agreement between the authors; the journal Deputy Editor, Yunfeng Lin; and John Wiley & Sons Ltd. Following additional research, the authors observed results that called into question their original findings. The retraction has been agreed due to a lack of sufficient data to support the article's conclusion.

C. Ji , J. Gao , and Y. Huang , “Legionella Pneumophila Infection Reduces the Mitochondrial Membrane Potential Through Lpg2444‐Inhibited Mitocytosis,” Cell Proliferation (Early View): 10.1111/cpr.13660.38764239

The above article, published online on 19 May 2024 in Wiley Online Library (wileyonlinelibrary.com), has been retracted by agreement between the authors; the journal Deputy Editor, Yunfeng Lin; and John Wiley & Sons Ltd. Following additional research, the authors observed results that called into question their original findings. The retraction has been agreed due to a lack of sufficient data to support the article's conclusion.

